# Australia

**Published:** 1896-12

**Authors:** 


### CONTROL WORK.

*Australia.* The prevalence of cattle-ticks in this country is causing deep concern among the owners of live-stock, and they are reaching out for methods for their control and extermination.

